# Long‐Term Functional Outcomes in Immunoglobulin‐Treated Multifocal Motor Neuropathy Evaluated Through the MMN‐Rasch‐Built Overall Disability Scale

**DOI:** 10.1111/jns.70079

**Published:** 2025-11-17

**Authors:** Muhammed A. Noushad, Ahmad Al‐Areed, Roshan Iqbal, Joumana Freiha, Chinar Osman, Yusuf A. Rajabally

**Affiliations:** ^1^ Wessex Neurological Centre University Hospital Southampton Southampton UK; ^2^ Inflammatory Neuropathy Service, Department of Neurology University Hospitals Birmingham Birmingham UK; ^3^ Aston Medical School Aston University Birmingham UK

**Keywords:** dose, immunoglobulin, minimal clinically important difference, MMN‐RODS, multifocal motor neuropathy, outcomes

## Abstract

**Background:**

Long‐term functional outcomes are uncertain in immunoglobulin‐treated multifocal motor neuropathy (MMN).

**Methods:**

We retrospectively studied consecutive subjects with MMN from two neuromuscular centres in Southampton and Birmingham, UK. Initial and latest MMN‐Rasch‐built Overall Disability Scale (MMN‐RODS) scores and latest immunoglobulin doses were collected. Latest dose alterations and resulting MMN‐RODS changes were ascertained.

**Results:**

We included 32 subjects with MMN (14 females and 18 males). Mean age was 60.0 years (SD: 11.7). Over a mean of 6.2 years, MMN‐RODS scores improved in 29 out of 32 (90.6%) subjects and worsened in 3 out of 32 (9.4%) subjects. Mean latest centile MMN‐RODS was improved compared to mean initial centile MMN‐RODS (81.53 [SD: 14.14] vs. 63.47 [SD: 13.82]; *p* < 0.001). Mean latest immunoglobulin dose was 26.3 g/week (range: 4–70). There were no associations of the latest immunoglobulin dose with age/disease duration/weight/gender/comorbidities/initial disability/latest disability. There were no inter‐centre differences in age/disease duration/weight/gender/comorbidities/initial disability/latest disability. The latest mean immunoglobulin dose was higher in Birmingham than in Southampton (33.9 g/week [SD: 17.1] vs. 18.8 g/week [SD: 8.0]; *p* = 0.004). Immunoglobulin dose dependency was observed in 16 out of 17 subjects whose last dose alteration was incremental, and in only 3 out of 15 subjects whose last dose alteration was decremental. Dose dependency was demonstrated in a greater proportion of subjects from Birmingham compared to Southampton (13/16 vs. 6/16; *p* = 0.03).

**Conclusions:**

Function as ascertained by the MMN‐RODS shows sustained improvement over > 6 years with individualised immunoglobulin dosing in most subjects with MMN. The large inter‐centre/inter‐individual dosing and dose dependency variations observed may suggest implications of patient‐ and physician‐related factors, which require further study.

## 1 Introduction

Multifocal motor neuropathy (MMN) is a treatable autoimmune neuropathy causing multifocal motor deficits affecting predominantly the muscles of the distal upper limbs [1, 2].

There is a paucity of data on long‐term outcomes in immunoglobulin‐treated MMN. To date, patient‐reported outcome measures (PROMs) have, to our knowledge, not been used in medium‐ to long‐term outcome evaluation. Several studies have suggested that, as a result of progressive axonal loss despite continuing immunoglobulin treatment, the prognosis of MMN after initial immunoglobulin response is one of progressive decline [3, 4, 5]. In a recent national survey, however, it was found that in 9 UK centres, physician‐assessed progression was favourable in > 85% of 92 treated subjects [6]. These subjects had, for the majority, been evaluated serially with the MMN‐Rasch‐built Overall Disability Scale (MMN‐RODS) [7], as opposed to what has been described in other cohorts through the use of strength measures. The mean dose of immunoglobulin used at our nine centres was otherwise found to be 30%–60% higher than in neighbouring countries, where a gradual decline was described, raising the question of the implication of dosage in maintaining clinical benefit. A similar hypothesis had been raised previously through the description of clinical stability in a small cohort of 11 subjects from the US centres, with continuing immunoglobulin doses comparable to those used at the 9 UK centres [8].

We conducted a retrospective study of subjects with MMN attending our two UK neuromuscular centres, with the principal aim to determine the long‐term prognosis of MMN in subjects on continuing immunoglobulin treatment using the MMN‐RODS. We have used since 2016, in our routine clinical practice, the MMN‐RODS, a disease‐specific PROM available since 2015, for monitoring of subjects with MMN [7]. The MMN‐RODS evaluates predominantly upper limb function through 25 questions, requiring standardised answers (‘easy to perform’, ‘difficult to perform’ and ‘impossible to perform’), producing a raw score ranging from 50 (*no disability*) to 0 (*maximal disability resulting in impossibility to perform all tasks*) [7]. This raw score is then transformed into a centile metric (algorithm kindly provided by Prof. Karin Faber, Maastricht University, Netherlands), as advised in the original publication. We also secondarily aimed (i) to calculate through distribution methods the minimal clinically important difference (MCID) for the centile MMN‐RODS score (ii) to determine long‐term prognosis evaluated through grip strength measurement, and its association with MMN‐RODS scores (iii) to ascertain mean immunoglobulin dose administered in our joint cohort, as well as in each centre in relation to baseline level and outcome (iv) to establish immunoglobulin dose dependency in our cohort and its subgroups through analysis of effects of the latest performed dose modification on the MMN‐RODS, and (v) to determine the eventual predictors of prognosis and immunoglobulin dosage in our cohort and its subgroups.

## 2 Materials and Methods

We included consecutive subjects with a clinical diagnosis of MMN attending University Hospital Southampton, UK, and University Hospitals Birmingham, UK between 2014 and 2025, meeting criteria for a diagnosis of definite, probable or possible MMN, as per European Federation of Neurological Societies/Peripheral Nerve Society (EFNS/PNS) 2010 Guidelines [9].

We collected demographic data, comorbidities, disease phenotype, disease duration, baseline centile MMN‐RODS at first evaluation with this scale, latest recorded centile MMN‐RODS, and the interval between initial and latest assessments. Available intermediate MMN‐RODS recordings (up to two for each subject) and their timings were collected to assess for longitudinal score changes. Baseline and latest (Jamar) grip strength measurement for the most affected hand was also collected. We recorded treatment protocols used, the latest immunoglobulin dosage administered and the mode of immunoglobulin administration. The MCID for the centile MMN‐RODS was determined through the ½ SD method. Proportions of subjects attaining MCID cut‐off levels for clinically meaningful amelioration were ascertained. Outcome comparisons were performed between subjects from our two centres. Associations were determined between outcome and the studied variables, including demographic, disease characteristics and immunoglobulin dosage. Immunoglobulin dose dependency was determined considering the latest dose alteration performed and the resulting proportions of subjects showing improvement vs. deterioration of the centile MMN‐RODS, in each studied subgroup and in combination.

Statistical analyses were performed with SPSS 28.0 (Armonk, USA). Comparison of proportions was performed by Fisher's exact tests, and comparison of means was performed by paired or independent *T*‐tests, as applicable. Associations were studied through Spearman's rank correlation. Independent associations were sought through linear regression. Significance was set at *p* < 0.05 for all tests.

This analysis was conducted as part of registered and approved retrospective clinical audits of the diagnosis and management of MMN at University Hospital Southampton, UK, and University Hospitals Birmingham, UK (Reg. no. SEV‐0890 and CARMS‐20703, respectively). Audit does not require Ethics Committee approval in the United Kingdom.

## 3 Results

### 3.1 Baseline Characteristics

Characteristics of the studied combined cohort are summarised in Table 1. We included 32 consecutive subjects with a diagnosis of MMN, meeting EFNS/PNS 2010 criteria. Sixteen subjects were recruited in Southampton and 16 in Birmingham. There were 14 females and 18 males (ratio: 1:1.29). Mean age was 60.0 years (SD: 11.6), mean age at onset was 47.7 years (SD: 11.0), mean latest recorded weight was 85.5 kg (SD: 20.7), and mean total disease duration was 149.8 months (SD: 80.4), or 12.5 years. Twenty‐nine subjects (90.6%) had pure asymmetrical upper limb disease, and three (9.4%) had asymmetrical mixed, upper and lower limb involvement. Twenty‐seven subjects (84.4%) had definite MMN, one (3.1%) had probable MMN, and four (12.5%) had possible MMN, as per EFNS/PNS 2010 criteria.

**TABLE 1:** Characteristics of 32 consecutive subjects with a diagnosis of MMN on individualised immunoglobulin treatment, from Southampton, UK and Birmingham, UK.

Mean age, years (SD)	60.0 (11.6)
Gender F:M (ratio)	14:18 (1:1.29)
EFNS/PNS 2010 MMN diagnostic sub‐category	– Definite: 27/32 (84.4%) – Probable: 1/32 (3.1%) – Possible 4/32 (12.5%)
MMN clinical phenotype	– Asymmetrical pure upper limb: 29/32 (90.6%) – Asymmetrical mixed upper/lower limb: 3/32 (9.4%)
Mean disease duration, months (SD)	149.8 (80.4)
Mean interval between initial and latest MMN‐RODS, months (SD)	74.6 (41.0)
Mean initial centile MMN‐RODS score (SD)	65.50 (9.50)
Mean latest centile MMN‐RODS score (SD)	81.53 (14.14)
Mean centile ∆MMN‐RODS (SD)	16.03 (13.28)
Clinically applicable distribution‐based MCID for centile MMN‐RODS	7
Modality of immunoglobulin administration	IVIg in 23/32 (71.9%)
	SCIg in 9/32 (28.1%)
Mean latest immunoglobulin dose, g/week (SD)	26.3 (15.0)

### 3.2 MMN‐RODS Evolution From Initial to Latest Assessment and MCID Derived by Distribution Method

The centile MMN‐RODS score improved by a mean of 16.03 points (SD: 13.28) from a baseline of 65.50 (SD: 9.50) to a latest recording of 81.53 (SD: 14.14) (*p* < 0.001), over a mean follow‐up period of 74.6 months (SD: 41.0), or 6.2 years.

The distribution‐based (½ SD) MCID for the centile MMN‐RODS was hence 6.64, and the clinically applicable MCID was 7 points. Twenty‐eight out of 29 subjects (96.6%) who improved on the centile MMN‐RODS reached the MCID.

### 3.3 Evolution of Grip Strength of the Most Affected Hand

From available data for 30 out of 32 (93.8%) subjects, the mean grip strength of the most affected hand improved by a mean of 4.7 kg (SD: 12.0) from a baseline of 16.2 kg (SD: 10.2) to a latest measurement of 20.8 kg (SD: 11.2) (*p* = 0.048), over a mean follow‐up period of 100.1 months (SD: 79.8), or 8.3 years.

The distribution‐based (½ SD) MCID was hence 6.0 kg. Fourteen out of 30 subjects (46.7%) who had serial grip strength assessments reached this MCID over the follow‐up period. The proportion of subjects attaining the MCID for grip strength was therefore lower than that attaining the MCID for the MMN‐RODS (14/30 vs. 28/32; *p* < 0.001).

### 3.4 Treatment Protocols, Latest Immunoglobulin Dose and Modality of Administration

Treatment protocols used in earlier disease stages varied between our two centres. In Southampton, after two initial doses of 2 g per kg of ideal body weight, the dose was decreased to 1 g per kg administered at an interval determined by the timing of relapse. Subsequent dose and frequency changes were decided on a case‐by‐case basis, depending on the maximal response attained and clinical stability. In Birmingham, a protocol used for chronic inflammatory demyelinating polyneuropathy (CIDP) was applied [10], with continuing doses of 2 g per kg of dosing weight every 4 weeks or earlier if relapse occurred, until the maximal response was obtained, following which the dose was reduced by 15%–25% intervals every few courses and the frequency adapted to the timing of wearing off of treatment effects, until the lowest effective dose and frequency were reached. Subsequent re‐increase in dose by similar percentages was performed in case of decline. The mode of administration was, as per our protocols, intravenous in all cases at initiation at both institutions, with variable timings for subcutaneous switch, when performed, predominantly conditioned by clinical stability, and at an equivalent mean weekly dose.

The mean latest immunoglobulin dose administered was 26.3 g per week (SD: 15.0, range: 4–70). Twenty‐three subjects (71.9%) were on intravenous immunoglobulin, and nine subjects (28.1%) were on subcutaneous immunoglobulin, at the latest assessment.

### 3.5 Associations of Latest Centile MMN‐RODS and Change in Centile MMN‐RODS (Centile ∆MMN‐RODS), With Demographic, Disease and Treatment Variables

The latest centile MMN‐RODS was associated with the latest grip strength of the most affected hand (*p* = 0.018), with younger current age (*p* = 0.02), with the initial centile MMN‐RODS (*p* = 0.03) and with female gender (*p* = 0.002). No association was found with age at disease onset (*p* = 0.16), weight (*p* = 0.43), disease duration (*p* = 0.33), latest immunoglobulin dose (*p* = 0.64) or mode of immunoglobulin administration (*p* = 0.62). An independent association of the latest centile MMN‐RODS was found with younger age (*p* = 0.043) and with female gender (*p* = 0.012), but not with the initial centile MMN‐RODS or the latest grip strength of the most affected hand.

The centile ∆MMN‐RODS was associated with younger age (*p* = 0.029), with the final centile MMN‐RODS (*p* < 0.001), with the latest grip strength (*p* = 0.041) and with the change in grip strength (*p* = 0.01). An independent association of the centile ∆MMN‐RODS was found with the latest centile MMN‐RODS (*p* < 0.001) and the change in grip strength (*p* = 0.008), the latter demonstrating concurrent improvement in the two scales in our cohort.

In the full cohort, the latest dose change was associated with the latest centile ∆MMN‐RODS (*p* = 0.013). In the 17 subjects whose latest dose change was an increase, 16 out of 17 improved on the centile MMN‐RODS (range: 3–21), whereas 1 out of 17 showed no change. The increase in immunoglobulin dose was directly associated with the latest centile ∆MMN‐RODS (*p* = 0.044), also consistent with the benefit of dose escalation. In the 15 subjects whose latest dose change was a decrease, 12 out of 15 showed no reduction of their centile MMN‐RODS, whereas 3 out of 15 worsened (range: −1 to −11). No association of the decrease in immunoglobulin dose was found with the latest centile ∆MMN‐RODS (*p* = 0.244), indicating the absence of deterioration with dose reduction in the cohort, as a whole.

### 3.6 Progression of Centile MMN‐RODS Scores Over the Course of the Study Period

In addition to the initial and latest centile MMN‐RODS recordings, we considered intermediate scores available in our cohort over the 74.6 months (6.2 years) follow‐up period. Data were available for one (31 out of 32 subjects) or two (29 out of 32 subjects) intermediate evaluations. The mean time from initial assessment to the first intermediate evaluation was 17.0 months (SD: 14.0). The mean time from initial assessment to the second intermediate evaluation was 54.1 months (SD: 27.6). The mean centile MMN‐RODS improved at the first intermediate evaluation compared to the initial assessment (74.7 [SD: 14.7] vs. 65.4 [SD: 9.6]; *p* < 0.001) but was comparable between the first and second intermediate evaluations (74.7 [SD: 14.7] vs. 77.4 [SD: 15.1]; *p* = 0.28). Further amelioration occurred between the second intermediate evaluation and the latest assessment (77.4 [SD: 15.1] vs. 81.1 [SD: 14.7]; *p* = 0.019). Hence, continuing improvement of the mean centile MMN‐RODS was observed in the whole cohort throughout the study period.

### 3.7 Inter‐Centre Differences in Demographics, Disease Characteristics, Outcome, Latest Immunoglobulin Dose and Immunoglobulin Dose Dependency

The findings are summarised in Table 2. There were no inter‐centre differences of current age (*p* = 0.90), age at onset (*p* = 0.67), gender distribution (*p* = 0.49), weight (*p* = 0.21), disease duration (*p* = 0.29), associated immunological comorbidities (*p* = 1), rate of concurrent diabetes (*p* = 0.33), initial centile MMN‐RODS (*p* = 0.095), latest centile MMN‐RODS (*p* = 0.37), centile ∆MMN‐RODS (*p* = 0.83) or interval between initial and latest MMN‐RODS scores (*p* = 0.83).

**TABLE 2:** Inter‐centre differences between 16 consecutive subjects with MMN on individualised immunoglobulin treatment, from Southampton, UK vs. 16 consecutive subjects with a diagnosis of MMN on individualised immunoglobulin treatment, from Birmingham, UK.

	Southampton	Birmingham	*p*
Mean age, years (SD)	59.7 (11.2)	60.3 (12.6)	0.90
Mean age at onset, years (SD)	48.6 (10.6)	46.8 (12.1)	0.67
Gender distribution F:M	6:10	8:8	0.49
Weight, kg (SD)	90.4 (21.5)	80.8 (20.2)	0.21
Disease duration, months (SD)	134.3 (85.8)	165.4 (77.0)	0.29
Immunological comorbidities	1/16	1/16	1.0
Concurrent diabetes	1/16	0/16	0.33
Mean initial centile MMN‐RODS (SD)	29.8 (8.5)	34.9 (7.4)	0.095
Mean latest centile MMN‐RODS (SD)	42.1 (9.8)	43.8 (6.0)	0.37
Mean centile ∆MMN‐RODS (SD)	12.4 (7.6)	8.8 (9.9)	0.83
Mean interval between MMN‐RODS, months (SD)	73.0 (45.5)	76.3 (39.0)	0.83
Mean latest immunoglobulin dose, g/week (SD)	18.8 (8.0)	33.9 (17.1)	***0.004***
Proportion of subjects on SCIg	4/16	5/16	1.0

*Note:* Statistical significance indicated by bold italics.

The interval between the initiation of the latest dose change and MMN‐RODS reassessment was comparable at both centres (mean 4.25 months [SD: 2.65] vs. mean 6.1 months [SD: 2.9]; *p* = 0.075). The direction of the latest dose change was comparable (Southampton: increase in 7 and reduction in 9 vs. Birmingham: increase in 10 and reduction in 6; *p* = 0.48). Mean latest dose changes were also comparable in Southampton and Birmingham (−2.12 g/week [SD: 7.78] vs. −0.16 g/week [SD: 11.8]; *p* = 0.59).

The latest immunoglobulin dose was higher in Birmingham compared to Southampton (33.9 g/week [SD: 17.1] vs. 18.8 g/week [SD: 8.0]; *p* = 0.004). The proportion of subjects receiving subcutaneous immunoglobulin treatment was identical (*p* = 1).

Comparative inter‐centre analyses of immunoglobulin dependency rates are shown in Table 3. Among subjects whose dose was increased, further improvement was observed as frequently in Southampton as in Birmingham (6/7 vs. 10/10; *p* = 0.41). Among subjects whose dose was reduced, deterioration occurred more frequently in Birmingham than in Southampton (3/6 vs. 0/9; *p* = 0.04). Hence, immunoglobulin dose dependency was overall demonstrated in a greater proportion of subjects in Birmingham than in Southampton (13/16 [81.25%] vs. 6/16 [37.5%]; *p* = 0.03).

**TABLE 3:** Analysis for immunoglobulin dose dependency in 16 consecutive subjects with MMN on individualised immunoglobulin treatment, from Southampton, UK and 16 consecutive subjects with a diagnosis of MMN on individualised immunoglobulin treatment, from Birmingham, UK.

	Southampton	Birmingham	*p*
Proportion of subjects improving on centile MMN‐RODS after an incremental latest dose change	6/7	10/10	*p* = 0.41
Proportion of subjects worsening on centile MMN‐RODS after a decremental latest dose change	0/9	3/6	***p = 0.04***
Overall proportion of subjects demonstrating immunoglobulin dose dependency through evaluation of effects of latest dose change on centile MMN‐RODS	6/16	13/16	***p = 0.03***

*Note:* Statistical significance indicated by bold italics.

## 4 Discussion

Despite an existing evidence base for immunoglobulin therapy for MMN [11], uncertainties have remained regarding its long‐term effectiveness. Several studies have reported a progressive decline despite continuing immunoglobulin treatment, predominantly using strength measures [3, 4, 5]. On the other hand, possible long‐term stability has previously rarely been described with the use of continuing high doses of immunoglobulin [8]. A recent multicentre analysis in the United Kingdom, where mean immunoglobulin doses are higher than in neighbouring countries, similarly suggested a favourable long‐term prognosis based on physician impression [6]. To date, no long‐term studies have, to our knowledge, been performed using it as a primary outcome. Individualised MCIDs for the MMN‐RODS have been derived from 26 patients, which computed the MCID‐SE (standard error) for each participant, and defined meaningful benefit by an MCID‐SE ≥ 1.96 [12]. To our knowledge, a distribution‐based MCID for the centile MMN‐RODS has not been proposed to date.

Our current retrospective analysis of 32 subjects with immunoglobulin‐treated MMN from our two UK neuromuscular centres shows sustained improvement of function as determined by amelioration of any amplitude of the centile MMN‐RODS, in > 90% of subjects, over > 6 years of follow‐up. Importantly, 28 out of 29 (96.6%) subjects who improved on the centile MMN‐RODS attained the distribution‐based MCID cut‐off of 7 points, derived from our cohort. The ∆MMN‐RODS correlated with improvement in grip strength measurements, although a significantly lower proportion of subjects attained the distribution‐based grip strength MCID cut‐off of 6 kg. This cut‐off for grip strength is, of note, close to that previously derived in subjects with CIDP [13, 14]. As may be expected, the latest recorded centile MMN‐RODS correlated with age, but also, unexpectedly, with female gender, suggesting a possible better prognosis in females with MMN. This finding requires further study in larger cohorts.

With reference to the long‐term functional improvement observed, we studied immunoglobulin dosing in our cohort, first by considering the latest dose used in each subject. This was highly variable between patients and was not associated with the latest centile MMN‐RODS or with the centile ∆MMN‐RODS achieved. These findings are consistent with previously reported large inter‐individual variability of immunoglobulin dosing requirements in MMN in other cohorts [15, 16], as well as in CIDP [17]. Of note, the mean latest dose was strikingly similar to that reported in the recent large UK MMN survey from nine centres (26.3 g/week [range: 4–70] vs. 26.2 g/week [range: 4–114]), indicating that our combined cohort was comparable in mean latest dosing to that used nationally [6]. We also considered immunoglobulin dose dependency and found a significant association with the benefit of the latest dose escalation in 17 subjects, but the absence of association with decline following the latest dose reduction in the remaining 15 subjects. We otherwise found that the latest dose of immunoglobulin administered was higher in Birmingham than in Southampton, despite comparable demographics, disease characteristics and mean initial centile MMN‐RODS, centile ∆MMN‐RODS and latest centile MMN‐RODS. As such, similar outcomes to those achieved in Southampton required higher immunoglobulin doses in Birmingham. However, and importantly, immunoglobulin dose dependency was demonstrated in 13 out of 16 (81.25%) of subjects from Birmingham, providing justification for the latest doses administered.

The precise reasons for the observed inter‐centre differences in dose and dose dependency are otherwise uncertain and may be multiple. Besides the well‐described large inter‐individual dosing‐requirement variations in MMN, and our small studied sample sizes increasing chances of random variability [18], these include effects of patient‐related covariates not evaluated in this study but of potential impact on PROM evaluation, such as socio‐economic status [19, 20], health literacy and numeracy [21, 22], patient perceptions of individual scale items [23] and psychiatric comorbidities [24, 25]. Similarly, physician‐related factors such as anchoring bias [26] and therapeutic inertia [27] may have had an effect on the practical application of treatment protocols, particularly in the absence of an MCID, and, consequently, on immunoglobulin dosing and dose dependency.

Our study has several limitations, including its retrospective design, small sample size, variable methods in PROM collection conducted partly through telephone consultations since the SARS‐CoV‐2 pandemic and non‐concurrent PROM and grip strength data collection. Immunoglobulin dosing protocols were furthermore dissimilar across our two centres. Furthermore, the MMN‐RODS was used over the period analysed, through raw score ascertainment without centile transformation and without an established MCID. Finally, the role of multiple other factors, both patient‐ and physician‐related, of possible impact on response and its measurement, as well as on immunoglobulin dosing, could not be considered.

In conclusion, the main findings of our analysis are of sustained, MCID‐attaining, functional benefit determined through the centile MMN‐RODS, over a mean of 6.2 years, in 87.5% of subjects with MMN treated with individualised immunoglobulin dosing. This challenges the current conventional belief of progressive decline in immunoglobulin‐treated MMN, suggested by few previous studies. The value of consistent higher dose immunoglobulin administration could not be demonstrated, but a large dosing range was observed, indicating wide inter‐individual variations of dosing requirements, for which no predictors could be ascertained. Further long‐term multicentre studies of outcome and its assessment methods, of immunoglobulin dosing, immunoglobulin dose dependency as well as their determinants, are required to further explore optimal treatment modalities and prognosis in MMN.

## Conflicts of Interest

Y.A.R. has received consultancy honoraria from Sanofi, Argenx, Janssen, LFB, Polyneuron, Grifols, Takeda, Dianthus and Vitaccess; has received educational sponsorships from LFB and CSL Behring; and has obtained research grants from LFB. C.O. has received speaker/consultancy honoraria from Takeda, Grifols and Terumo BCT. The other authors declare no conflicts of interest.

## Data Availability

The data that support the findings of this study are available on request from the corresponding author. The data are not publicly available due to privacy or ethical restrictions.
